# Disease Prevention: Saving Lives or Reducing Health Care Costs?

**DOI:** 10.1371/journal.pone.0104469

**Published:** 2014-08-12

**Authors:** Inge Grootjans-van Kampen, Peter M. Engelfriet, Pieter H. M. van Baal

**Affiliations:** 1 Education and Student Affairs, Technical University, Delft, The Netherlands; 2 Centre for Prevention and Health Services Research, Institute for Public Health and the Environment, Bilthoven, The Netherlands; 3 Institute of Health Policy and Management, Erasmus University, Rotterdam, The Netherlands; Old Dominion University, United States of America

## Abstract

**Background:**

Disease prevention has been claimed to reduce health care costs. However, preventing lethal diseases increases life expectancy and, thereby, indirectly increases the demand for health care. Previous studies have argued that on balance preventing diseases that reduce longevity increases health care costs while preventing non-fatal diseases could lead to health care savings. The objective of this research is to investigate if disease prevention could result in both increased longevity and lower lifetime health care costs.

**Methods:**

Mortality rates for Netherlands in 2009 were used to construct cause-deleted life tables. Data originating from the Dutch Costs of Illness study was incorporated in order to estimate lifetime health care costs in the absence of selected disease categories. We took into account that for most diseases health care expenditures are concentrated in the last year of life.

**Results:**

Elimination of diseases that reduce life expectancy considerably increase lifetime health care costs. Exemplary are neoplasms that, when eliminated would increase both life expectancy and lifetime health care spending with roughly 5% for men and women. Costs savings are incurred when prevention has only a small effect on longevity such as in the case of mental and behavioural disorders. Diseases of the circulatory system stand out as their elimination would increase life expectancy while reducing health care spending.

**Conclusion:**

The stronger the negative impact of a disease on longevity, the higher health care costs would be after elimination. Successful treatment of fatal diseases leaves less room for longevity gains due to effective prevention but more room for health care savings.

## Introduction

Worldwide ageing of populations is perceived as a threat to the global economy. One major consequence of ageing is claimed to be a rising proportion of GDP spent on health care. Population ageing refers to an increase in the number of old persons relative to the number of young persons, especially those aged 80 and above [1]. The main causes of population ageing are a decrease in fertility and an increase in life expectancy (increased longevity). To counter growing health care costs from ageing, it has been put forward that disease prevention might decrease disease-related costs [2]. Indeed, preventing a disease would avoid costs aimed at that particular disease and therefore could result in decreased health care costs in the short run. However, prevention of some diseases will increase longevity. Then, people will (ceteris paribus) consume more health care since the additional years are, on average, spent in less than ideal health [3]. To what extent the additional health care costs in ‘added’ life years outweigh savings in ‘normal’ years differs per disease and type of preventive intervention [4], [5]. Diseases associated with a high mortality risk have a strong negative impact on longevity, especially if these diseases have an early age of onset. The situation is different with chronic or disabling diseases [6]. Persons who attract such diseases can often live close to the average life expectancy. Nevertheless, chronic diseases may cause a reduction in quality of life and bring about continuous need for health care services.

Two decades ago, Bonneux and colleagues [7] investigated what would happen to Dutch expected lifetime health care costs in the hypothetical situation that certain diseases are eliminated. They found an inverse relation between degree of fatality and costs. This implies that disease-related costs for highly fatal diseases, such as coronary heart disease and cancer, account for only a small percentage of total health care costs, while diseases with a low mortality rate, such as mental disorders, account for a substantial part of the allocated health care budget. The study concluded that in countries with low mortality rates, elimination of fatal diseases through successful prevention increases health care costs due to medical expenses during life years gained. These findings indicated that prevention targeted at risk factors related to lethal diseases such as smoking, and to a lesser extent obesity, might not result in savings in medical costs [4], [5]. Since Bonneux and colleagues' study three important variables have changed. First, the statistics of primary causes of death have changed. Compared to two decades ago, mortality rates of the Dutch population have declined [8]. More specifically, mortality rates from coronary heart disease have halved for both sexes [9]; for patients admitted with myocardial infarction mortality rates have gone down by two-thirds [10]. Second, the Dutch costs of illness have risen each year [11]. Third, new insights into the effect of increased longevity on health costs have been developed. Traditionally, it was believed that increased longevity would strongly increase the demand for health care as average health care costs rise sharply with age. However, focussing only on age, sex and disease ignores a crucial element know as proximity or time to death. Zweifel and colleagues [1], and later Seshamani and Gray [12], explain that proximity to death is more crucial to the level of individual medical costs than age. The terminal phase of life is expensive whenever it occurs. As mortality increases with age, average health care costs also increase with age. However, costs per care provider category are affected differently by the effect of time to death. Long-term care costs for people who already make use of this type of care increase with age, regardless of time to death [13]. Also, the effect of time to death on health care costs differs per disease and is strongest for most types of neoplasms [14].

This study investigates the differential effects of prevention on longevity and lifetime health care costs. In particular, it sheds light on the relationship between disease-fatality and the potential of prevention to lower health care costs. We adopt a similar approach to Bonneux and colleagues, using more recent mortality and cost data and take proximity to death into account

## Methods

This research used life table techniques to calculate life expectancy (LE), both standard LE (including all causes of death) and cause-deleted LE (where specific causes of death are deleted) in relation to lifetime health care costs. Cause-deleted life tables show what a cohort's life expectancy and associated health care costs would be when no person dies from a particular disease (Primary Cause of Death (PCoD). In total 24 life tables were constructed for different diseases and disease categories. Data on mortality and population size was collected from Statline, the online database from Statistics Netherlands. To compare the results of this study to those of Bonneux and colleagues [7], we used the ICD-9 codes of the PCoDs from Statline to combine the primary causes into (mutually exclusive) disease categories. Similar to Bonneux and colleagues we used the disease categories of the Cost of Illness (COI) studies. In those studies total direct health care costs in different health care settings in the Netherlands are uniquely attributed to 107 diseases specified by gender and age [15]. For comparative purposes, a number of single diseases were added that were also incorporated by Bonneux and colleagues.

Estimates of life expectancy and lifetime health care costs from the cause-deleted life tables were compared to the all cause life table. Although complete elimination of a disease is almost never attainable, the relevant mechanisms of disease prevention are the same whether or not elimination is total or partial. Moreover, it enhances the visibility of the mechanisms at work as well as the ease of calculation, since cause-specific mortality then is zero.

Mortality probabilities denoted *q* in the absence of disease(s) *z*, needed to construct cause deleted life expectancy were calculated in the following manner:
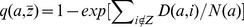
(1)Where 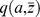 is the mortality probability at age *a* in the absence of disease (group) z, *i* an index for diseases and *D(a,i)* the number of deaths from cause *i* at age *a* and *N(a)* the average population at age *a*. Data on the number of deaths was available in (5-year) age intervals for the year 2009 [16]. In order to construct a full life table and to retain most data precision, the cause specific mortality data had to be converted into single-year mortality rates.

Average annual health care costs *c* at age *a* in the absence of disease (group) *z* were calculated in the following manner;
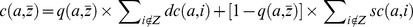
(2)


Where 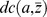 denotes average health care costs at age *a* for those who die at that age (excluding costs associated with disease group *z*) and, similarly, 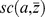 denotes average annual health care costs for those who survive at age *a* (again excluding costs associated with disease group z).

Equation (2) shows that average costs 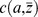 are a weighted average of the costs of people who die at a particular age and those who die at a later age. Elimination of a disease affects average health care costs at a given age in two different ways. First, it affects the sums of 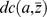 and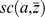 over all diseases as the costs of that disease (group) are excluded. Second, there is an indirect effect through the influence on 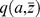.As costs in the last year of life 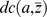 are higher than in other years, a decrease in the mortality probability due to elimination of a disease decreases average health care costs at that age. However, as the relation between health care costs and the last year of life differs per disease there might be complex interactions.

Data on health care costs 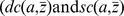 came from software package PAID 1.0 (Practical Application to Include Disease Costs) [17]. PAID is a toolkit that enables researchers to estimate future medical costs by calculating annual per capita health care costs stratified by disease, age, sex and proximity to death. As a backbone for PAID 1.0, data from the Costs of Illness (COI) study for the Netherlands from were used [15]. In that study total direct health care costs in different health care settings in the Netherlands in 2005 are uniquely attributed to 107 diseases specified by gender and age.The COI study includes spending on ambulatory care, hospital care, medication as well as spending on long term care. Using data from other studies [14] these annual age, gender and disease specific health care expenditures per capita are partitioned into annual per capita expenditure in the last year of life and all other years for all diseases (for details see [17]). PAID 1.0 consists of a series of worksheets in Excel in which diseases can be selected (free download at www.bmg.eur.nl/personal/vanbaal/paid.http). Cause deleted life expectancy 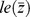 and lifetime health care costs 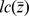 were then estimated in the following manner: 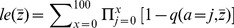
(3)

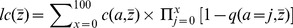
(4)


## Results

In this paper, for legibility, only the results for the most noticeable disease categories and one single disease, relevant for the discussion, are shown (the full tables are presented as supplementary material: Table S1 contains all results for men and Table S2 contains all results for women). The tables, one for men (table 1) and one for women (table 2), include all values of, as well as absolute and relative changes in, life expectancy and lifetime health care costs, subdivided into health care sectors such as hospital care, as well as the aggregate.

**Table 1:** Overview results disease elimination on life expectancy and health care costs for men.

Disease-category eliminated	Life expectancy at birth / absolute difference compared to base case / relative difference compared to base case	Lifetime health care costs ×1000 Euros / absolute difference compared to base case / relative difference compared to base case					
		All health care providers combined	Hospitals	Nursing and residential care facilities	Providers of ambulatory health care	Retail sale and other providers of medical goods	Other health care providers
None (base case)	78.5	250	98	33	50	39	29
Neoplasms	82.6/+4.1/+5.2%	266/+16.3/+6.5%	96/−1.7/−1.7%	44/+10.2/+30.5%	53/+3.3/+6.6%	42/+3.1/+7.9%	30/+1.5/+5.1%
Mental and behavioral disorders	78.8/+0.3/+0.3%	213/−36.7/−14.7%	82/−16.3/−16.6%	17.6/−15.9/−47.4%	48/−1.5/−3.1%	38/−1.1/−2.8%	27/−1.9/−6.4%
Diseases of the circulatory system	81.6/+3.0/+3.9%	240/−12.6/−5.1%	86/−12.1/−2.3%	37/+3.1/+9.3%	49/−1.0/−1.9%	37/−2.4/−6.0%	28/−0.3/−1.1%
Coronary Heart Disease	79.5/+0.9/+1.2%	246/−3.3/−1.3%	94/−4.4/−4.5%	36/+2.4/+1.2%	50/−0.1/−0.2%	38/−1.1/−2.7%	29/−0.2/−0.6%
Diseases of the digestive system	83.0/+0.4/+0.4%	305/−20.6/−6.3%	100/−5.7/−5.4%	69/+1.5/+2.2%	59/−11.8/−16.6%	46/−3.3/−6.6%	31/−1.3/−4.2%

**Table 2:** Overview results elimination of diseases on life expectancy and health care costs for women.

Disease−category eliminated	Life expectancy at birth / absolute difference compared to base case / relative difference compared to base case	Lifetime health care costs ×1000 Euros / absolute difference compared to base case / relative difference compared to base case					
		All health care providers combined	Hospitals	Nursing and residential care facilities	Providers of ambulatory health care	Retail sale and other providers of medical goods	Other health care providers
None (base case)	82.6	326	106	68	71	49	32
Neoplasms	86.3/+3.6/+4.4%	342/+16.9/+5.2%	102/−3.8/−3.6%	82/+14.4/+21.2%	74/+2.9/+4.0%	52/+3.0/+6.2%	33/+0.5/+1.6%
Mental and behavioral disorders	83.2/+0.5/+0.6%	268/−58.2/−17.8%	89/−16.6/−15.7%	32/−35.6/−52.5%	69/−2.0/−2.8%	48/−1.5/−3.1%	30/−2.5/−7.9%
Diseases of the circulatory system	85.6/+2.9/+3.5%	324/−1.9/−0.6%	98/−8.1/−7.7%	76/+8.5/+12.6%	71/0/0%	47/−2.4/−4.8%	32/+0.1/+0.3%
Coronary Heart Disease	83.2/+0.6/+0.7%	326/+0.6/+0.2%	104/−2.0/−1.9%	71/+3.1/+4.6%	71/+0.3/+0.4%	49/−0.8/−1.5%	32/0/0%
Diseases of the digestive system	83.0/+0.4/+0.4%	305/−20.6/−6.3%	100/−5.7/−5.4%	69/+1.5/+2.2%	59/−11.8/−16.6%	46/−3.3/−6.6%	31/−1.3/−4.2%

Taking the all-cause life table as a base case, the disease categories that would result in the greatest increases in longevity, if eliminated, were for both sexes “neoplasms” (+4.1 and +3.6 years for men and women respectively) and “diseases of the circulatory system” (+3.0 and +2.9 years for men and women respectively).

The disease category that would result in lowest lifetime health care costs if eliminated is (with the values in thousands of Euros in parentheses) “mental and behavioural disorders” (€36,700 and €58,200 lower for men and women respectively). The only category that would result in higher lifetime health care costs if eliminated is “neoplasms” (€16,300 and €16,900 higher for men and women respectively). However, at the level of subcategories, we also identified a disease category which, if eliminated, would result in higher lifetime health care costs: coronary heart disease for women (− €3,300 and +€600 for men and women respectively). This finding will be explained in the discussion section.

The cost results have thus far been described in aggregate. More insight into the mechanisms through which prevention impacts on health care costs is gained by considering the distribution of costs over the different provider categories. Elimination of highly fatal diseases, such as ‘neoplasms’, results in a decrease in “hospital care” costs, but a large increase in “nursing and residential care facilities”. In contrast, eliminating “mental and behavioural disorders” would result in savings both in “hospital care” and “nursing and residential care facilities”. Costs on “nursing home and residential care” increase with elimination of acute and fatal diseases (e.g. neoplasms), and decreases with chronic and less fatal diseases (e.g. mental and behavioural disorders). Costs on “providers of ambulatory health care”, “retail sale and other providers of medical goods” and “other health care providers” changed less than the other two provider categories.

## Discussion

The results of this research show that elimination of fatal diseases would lead to increased longevity as well as higher lifetime health care costs. Thereby, our findings confirm for a large part the results of the study of Bonneux and colleagues [7]: in the Netherlands, elimination of fatal diseases would increase health care costs and elimination of non-fatal diseases would lower lifetime health care costs. However, there are some differences in our findings due to temporal changes in primary causes of death and costs of illness. For instance, as costs of mental and behavioural disorders have risen, potential savings have increased. Moreover, we also used a somewhat different method to estimate lifetime health care costs, taking into account research emphasizing the relevance of time to death.

Most important, Bonneux and colleagues found that elimination of “diseases of the circulatory system” would result in higher lifetime health care costs (+5.2% for men and +10.7% for women) and in our study that would result in lower costs (−5.1% for men and −1.9% for women). Also, they found increases in longevity with that elimination as great as a 7.1% increase for men and a 6.3% increase for women, though in this research it resulted in more moderate increases of 3.0% and 2.9% for men and women respectively.

The changes over time indicate that potential increases in longevity decrease with elimination of diseases of the circulatory system, whereas lifetime health care costs saw a change from higher to lower costs. This can be explained by a change on the scale from acute to chronic: the availability of more effective treatments for circulatory diseases has resulted in increased survival (and reduced marginal gains), but also higher costs. Successful treatment of fatal diseases thereby diminishes the potential for achieving increases in longevity by prevention, while at the same time it creates opportunities to spend less on health care.

The added value of the results of this research lies in a number of areas. First, this study was built on more recent Dutch epidemiological and cost data, providing an up to date view on life expectancy and lifetime health care costs in the Netherlands. Second, a comprehensive range of diseases and disease categories were included, making comparisons possible both across diseases and across time. Third, as mentioned above, the relevance of time to death was explicitly dealt with in the methods of calculation. In a sensitivity analysis we calculated lifetime health costs without incorporating time to death. By neglecting the mechanism that for most diseases health care expenditures are concentrated in the last year of life two diseases (COPD and coronary heart disease for women) appeared to induce lower costs if eliminated.

By assuming the improbable situation of total elimination of diseases, insights can be gained into what the relative benefits and consequences could be of investing in the prevention of particular diseases and disease categories. In this regard the gains in life expectancy as well as the cost implications calculated with the life tables are at the extremes of the spectrum of what effective preventative interventions can achieve. The results highlight that the lethality of a disease is a crucial factor influencing these variables. In this respect, an important potential confounding factor should be mentioned, namely the uncertainty surrounding the attribution of a specific cause of death. When physicians determine the primary cause of death, this is often not based on a full-scale autopsy. This gives room to differences in interpretations and confounds the validity of the data. Also, it is often difficult to determine ‘the real’ cause of death, when several interrelated potential causes are at work, such as in the case of a complex disease such as heart failure [18]. More importantly, many preventive interventions are not targeted at single diseases, but rather at risk factors that are related to a variety of diseases. Consequently, results for specific intervention measures would depend on that mix [5]. Although our findings facilitate a better understanding of these interventions, separate analyses should be carried out to quantify gains. While a risk factor like smoking is strongly related to several lethal diseases, this is not the case for a risk factor like obesity that is also related to non-fatal diseases that are highly prevalent.

A potential limitation of this study is that we could not take into health care costs outside of the health care setting, which might have biased our findings. In particular, for instance treatment for mental health problems could save on long term healthcare expenditures with only a small effect on life expectancy. It should be noted, however, that the expenditure data we used include expenditures on long term care facilities. As in the Netherlands, long term care facilities are quite generous in comparison with other developed countries and to a large extent publicly financed [19], we do not think that including health care spending outside the health care setting would have a big impact on our findings. Out-of-pocket health care spending is very low in the Netherland compared to other countries. Only spending on informal care is not included in our calculations. However, as the level of formal care is quite high in the Netherlands we expect the influence of this to be limited. Yet, this limitation should be taken into account when extrapolating the results to health care systems in other countries.

The findings of this study lead us to three concluding remarks. First, the results can help policy makers and researchers evaluate the possible effects of preventive measures on both longevity and health care costs. Second, the evolution of diseases of the circulatory system such as coronary heart disease, from a disease (category) that, if eliminated, would increase lifetime health care costs to one that would decreases it, points to a very important mechanism mediating the effects of disease prevention. There is an interaction between preventive and curative healthcare. The more effective curative care is, the more it reduces the potential health gains of preventative care. On the other hand, it simultaneously opens a window of opportunity for reducing health care costs. This should make prevention a very interesting policy aim and also a profitable direction for insurers [20]. For researchers the interaction between preventive and curative care is relevant for correctly estimating lifetime healthcare costs because otherwise the effect of prevention on cost-effectiveness may be overestimated. For example, Lansdorp-Vogelaar and colleagues [21] assume that costs of treating colorectal cancer will increase and therefore prevention becomes more cost effective. However, rising investments on curative interventions for colorectal cancer improve the prospects of patients and therewith decrease the potential health gains of prevention. This leads to a new paradox: successful treatment of fatal diseases leaves less room for life expectancy gains due to effective prevention but more room for health care savings. On a third and final note, increasing longevity is not the only aim of disease prevention. The other very important goal is improving quality of life. Therefore, it would be interesting to perform additional research that includes measures of quality of life into the life tables. This would give a more complete picture of the mechanisms of disease prevention and its potential effects. Especially for non-fatal diseases health benefits of prevention are underestimated if one focuses on length of life only and ignores benefits in terms of quality of life. However, here, we expect similar mechanisms as with health care expenditures. While for diseases that do not strongly decrease longevity it suffices to look at quality of life losses for that disease only, in the case of lethal diseases it is important to look both at quality of life losses and life years lost.

Concluding, the stronger the negative impact of a disease on longevity, the higher health care costs would be after hypothetical elimination of that disease. Successful treatment of fatal diseases leaves less room for longevity gains due to effective prevention, but more room for health care savings.

## Supporting Information

Table S1 **Overview results disease elimination on life expectancy and health care expenditure for men.**
(DOCX)

Table S2 **Overview results disease elimination on life expectancy and health care expenditure for women.**
(DOCX)
