# Supplementary material for: Disease Prevention: Saving Lives or Reducing Health Care Costs?
Source: PLoS One. 2014 Aug 12;9(8):e104469. doi: 10.1371/journal.pone.0104469 (PMC4130534; doi:10.1371/journal.pone.0104469)
Supplement: Table S1 — Overview results disease elimination on life expectancy and health care expenditure for men. (DOCX) [file pone.0104469.s001.docx]

| **Table S1: Overview results disease elimination on life expectancy and health care expenditure for men** | | | | | | | |
| --- | --- | --- | --- | --- | --- | --- | --- |
| **Disease-category eliminated** | **Life expectancy at birth**  **(absolute & relative difference from base case)** | **Lifetime health care expenditures (x1000 Euros)** | | | | | |
|  |  | **All health care providers combined** | **Hospitals** | **Nursing and residential care facilities** | **Providers of ambulatory health care** | **Retail sale and other providers of medical goods** | **Other health care providers** |
| None (base case) | 78.5 | 250 | 98 | 33 | 50 | 39 | 29 |
| Neoplasms | 82.6  (+ 4.1)  (+ 5.2%) | 266  (+ 16.3)  (+ 6.5%) | 96  (- 1.7)  (- 1.7%) | 44  (+ 10.2)  (+ 30.5%) | 53  (+ 3.3)  (+ 6.6%) | 42  (+ 3.1)  (+ 7.9%) | 30  (+ 1.5)  (+ 5.1%) |
| *malignant a la Bonneux^[[1]](#footnote-1)^* | *82.6*  *(+ 4.1)*  *(+ 5.1%)* | *267*  *(+ 17.5)*  *(+ 7.0%)* | *98*  *(- 0.5)*  *(- 0.5%)* | *43*  *(+ 9.9)*  *(+ 29.5%)* | *53*  *(+ 3.5)*  *(+ 7.0%)* | *42*  *(+ 3.0)*  *(+ 7.7%)* | *30*  *(+ 1.5)*  *(+ 5.4%)* |
| *Neoplasms breastcancer* | *78.5*  *(+ 0.0)*  *(+ 0.0%)* | *250*  *(+ 0.0)*  *(+ 0.0%)* | *98*  *(+ 0.0)*  *(+ 0.0%)* | *33*  *(+ 0.0)*  *(+ 0.0%)* | *50*  *(+ 0.0)*  *(+ 0.0%)* | *39*  *(+ 0.0)*  *(+ 0.0%)* | *29*  *(+ 0.0)*  *(+ 0.0%)* |
| *Neoplasms lungcancer* | *79.5*  *(+ 1.0)*  *(+ 1.3%)* | *256*  *(+ 6.2)*  *(+ 2.5%)* | *99*  *(+ 0.8)*  *(+ 0.8%)* | *36*  *(+ 2.4)*  *(+ 7.3%)* | *51*  *(+ 1.2)*  *(+ 2.5%)* | *41*  *(+ 1.2)*  *(+ 3.0%)* | *29*  *(+ 0.5)*  *(+ 1.8%)* |
| *Neoplasms colorectal cancer* | *78.9*  *(+ 0.4)*  *(+ 0.5%)* | *251*  *(+ 1.0)*  *(+ 0.4%)* | *97*  *(- 0.8)*  *(- 0.8%)* | *34*  *(+ 1.0)*  *(+ 3.0%)* | *50*  *(+ 0.3)*  *(+ 0.5%)* | *40*  *(+ 0.5)*  *(+ 1.2%)* | *29*  *(+ 0.1)*  *(+ 0.4)* |
| Mental and behavioral disorders | 78.8  (+ 0.3)  (+ 0.3%) | 213  (- 36.7)  (- 14.7%) | 82  (- 16.3)  (- 16.6%) | 17.6  (- 15.9)  (- 47.4%) | 48  (- 1.5)  (- 3.1%) | 38  (- 1.1)  (- 2.8%) | 27  (- 1.9)  (- 6.4%) |
| Infectious and parasitic disease | 78.7  (+ 0.1)  (+ 0.2%) | 243  (- 6.7)  (- 2.7) | 97  (- 1.2)  (- 1.2%) | 34  (+ 0.2)  (+ 0.5%) | 48  (- 1.4)  (- 2.9%) | 37  (- 2.3)  (- 6.0%) | 27  (- 1.9)  (-6.6%) |
| Endocrine, nutritional and metabolic | 78.8  (+ 0.2)  (+ 0.3%) | 245  (- 4.4)  (- 1.8%) | 97  (- 1.5)  (- 1.5%) | 34  (+ 0.2)  (+ 0.5%) | 49  (- 0.7)  (- 1.4%) | 37  (- 2.1)  (- 5.4%) | 29  (- 0.3)  (- 1.0%) |
| Diseases of the blood and the blood-forming organs | 78.6  (+ 0.0)  (+ 0.0%) | 249  (- 1.0)  (- 0.4%) | 97  (- 0.7)  (- 0.7%) | 33  (+ 0.0)  (+0.0%) | 50  (- 0.2)  (- 0.4%) | 39  (- 0.1)  (- 0.3%) | 29  (- 0.1)  (- 0.2%) |
| Diseases of the circulatory system | 81.6  (+ 3.0)  (+ 3.9%) | 240  (- 12.6)  (- 5.1%) | 86  (- 12.1)  (- 12.3%) | 37  (+ 3.1)  (+ 9.3%) | 49  (- 1.0)  (- 1.9%) | 37  (- 2.4)  (- 6.0%) | 28  (- 0.3  (- 1.1%) |
| *Coronary Heart Disease* | *79.5*  *(+ 0.9)*  *(+ 1.2%)* | *246*  *(- 3.3)*  *(- 1.3%)* | *94*  *(- 4.4)*  *(- 4.5%)* | *36*  *(+ 2.4)*  *(+ 1.2%)* | *50*  *(- 0.1)*  *(- 0.2%)* | *38*  *(- 1.1)*  *(- 2.7%)* | *29*  *(- 0.2)*  *(- 0.6%)* |
| *Stroke* | *79*  *(+ 0.5)*  *(+ 0.6%)* | *244*  *(- 5.1)*  *(- 2.1%)* | *96*  *(- 1.9)*  *(- 1.9%)* | *30*  *(- 3.5)*  *(- 10.5%)* | *50*  *(- 0.0)*  *(- 0.0%)* | *40*  *(+ 0.3)*  *(+ 0.9%)* | *29*  *(- 0.0)*  *(- 0.2%)* |
| Diseases of the nervous system | 78.9  (+ 0.3)  (+ 0.4%) | 233  (- 16.5)  (- 6.6%) | 92  (- 6.0)  (- 6.2%) | 32  (- 1.6)  (- 4.8%) | 48  (- 2.2)  (- 4.4%) | 34  (- 5.6)  (- 14.2%) | 28  (- 1.0)  (- 3.6%) |
| Diseases of the respiratory system | 79.5  (+ 1.0)  (+ 1.3%) | 244  (- 5.6)  (- 2.2%) | 94  (- 4.1)  (- 4.2%) | 36  (+ 2.2)  (+ 6.5%) | 49  (- 1.1)  (- 2.3%) | 37  (- 2.3)  (- 5.8%) | 29  (- 0.2)  (- 0.8%) |
| *COPD* | *79.0*  *(+ 0.5)*  *(+ 0.6%)* | *248*  *(- 1.3)*  *(- 0.5%)* | *97*  *(- 1.0)*  *(- 1.0%)* | *35*  *(+ 1.1)*  *(+ 3.4%)* | *50*  *(+ 0.0)*  *(+ 0.0%)* | *38*  *(- 1.4)*  *(- 3.6%)* | *29*  *(- 0.0)*  *(- 0.1%)* |
| Diseases of the digestive system | 78.9  (+ 0.4)  (+ 0.4%) | 231  (- 18.9)  (- 7.6%) | 92  (- 5.8)  (- 5.9%) | 34  (+ 0.6)  (+ 1.7%) | 40  (- 9.9)  (- 19.9%) | 37  (- 2.6)  (- 6.5%) | 28  (- 1.2)  (- 4.2%) |
| Diseases of the genitourinary system | 78.7  (+ 0.2)  (+ 0.2%) | 243  (- 6.5)  (- 2.6%) | 95  (- 3.3)  (- 3.4%) | 34  (+ 0.3)  (+ 1.0%) | 49  (- 1.0)  (- 1.9%) | 37  (- 1.9)  (- 4.8%) | 28  (- 0.7)  (- 2.3%) |
| Pregnancy, childbirth and the puerperium | 78.5  ( 0)  ( 0) | 249  (- 0.7)  (- 0.3%) | 98  (- 0.4)  (- 0.4%) | 33  ( 0)  ( 0) | 50  (- 0.2)  (- 0.4%) | 39  ( 0)  ( 0) | 29  (- 0.1)  (- 0.3%) |
| Diseases of the skin and subcutaneous tissue | 78.5  (+ 0.0)  (+ 0.0) | 245  (- 4.2)  (- 1.7%) | 96  (- 1.8)  (- 1.9%) | 33  (- 0.1)  (- 0.2%) | 49  (- 1.0)  (- 2.1%) | 38  (- 1.0)  (- 2.6%) | 29  (- 0.3)  (- 0.9%) |
| Diseases of the musculoskeletal system and connective tissue | 78.6  (+ 0.0)  (+ 0.0%) | 235  (- 14.9)  (- 6.0%) | 92  (- 6.6)  (- 6.7%) | 33  (- 0.4)  (- 1.1%) | 45  (- 5.1)  (- 10.2%) | 37  (- 2.0)  (- 5.0%) | 28  (- 1.0)  (- 3.3%) |
| Certain conditions in the perinatal period | 78.7  (+ 0.2)  (+ 0.2%) | 247  (- 2.3)  (- 0.9%) | 97  (- 1.5)  (- 1.6%) | 34  (+ 0.1)  (+ 0.2%) | 49  (- 0.6)  (- 1.2%) | 39  (+ 0.1)  (+ 0.2%) | 28  (- 0.4)  (- 1.3%) |
| Symptoms, signs and abnormalities | 78.9  (+ 0.4)  (+ 0.5%) | 228  (- 21.5)  (- 8.6%) | 89  (- 9.7)  (- 9.8%) | 34  (+ 0.6)  (+ 1.9%) | 44  (- 5.9)  (- 11.9%) | 34  (- 5.3)  (- 13.4%) | 28  (- 1.3)  (- 4.4%) |
| Injury, poison and others | 79.3  (+ 0.8)  (+ 1.0%) | 245  (- 4.5)  (- 1.8%) | 94  (- 3.9)  (- 4.0%) | 33  (- 0.0)  (- 0.1%) | 49  (- 1.0)  (- 1.9%) | 40  (+ 0.6)  (+ 1.5%) | 29  (- 0.2)  (- 0.7%) |
| *Traffic accident* | *78.7*  *(+ 0.2)*  *(+ 0.2)* | *-* | *-* | *-* | *-* | *-* | *-* |

1. *Italics* and right text alignment indicate sub categories [↑](#footnote-ref-1)
