# Supplementary material for: Disease Prevention: Saving Lives or Reducing Health Care Costs?
Source: PLoS One. 2014 Aug 12;9(8):e104469. doi: 10.1371/journal.pone.0104469 (PMC4130534; doi:10.1371/journal.pone.0104469)
Supplement: Table S2 — Overview results disease elimination on life expectancy and health care expenditure for women. (DOCX) [file pone.0104469.s002.docx]

| **Table S2: Overview results elimination of diseases on life expectancy and health care expenditure for women** | | | | | | | |
| --- | --- | --- | --- | --- | --- | --- | --- |
| **Disease-category eliminated** | **Life expectancy at birth**  **(absolute & relative difference from base case)** | **Lifetime health care expenditures (x1000 Euros)** | | | | | |
|  |  | **All health care providers combined** | **Hospitals** | **Nursing and residential care facilities** | **Providers of ambulatory health care** | **Retail sale and other providers of medical goods** | **Other health care providers** |
| None (base case) | 82.6 | 326 | 106 | 68 | 71 | 49 | 32 |
| Neoplasms | 86.3  (+ 3.6)  (+ 4.4%) | 342  (+ 16.9)  (+ 5.2%) | 102  (- 3.8)  (- 3.6%) | 82  (+ 14.4)  (+ 21.2%) | 74  (+ 2.9)  (+ 4.0%) | 52  (+ 3.0)  (+ 6.2%) | 33  (+ 0.5)  (+ 1.6%) |
| *Neoplasms malignant a la Bonneux^[[1]](#footnote-1)^* | *86.2*  *(+ 3.6)*  *(+ 4.3%)* | *346*  *(+ 20.0)*  *(+ 6.1%)* | *104*  *(- 1.7)*  *(- 1.7%)* | *82*  *(+ 14.6)*  *(+ 21.5%)* | *74*  *(+ 3.4)*  *(+ 4.8%)* | *52*  *(+ 3.1)*  *(+ 6.2%)* | *33*  *(+ 0.7)*  *(+ 2.2%)* |
| *Neoplasms breastcancer* | *83.2*  *(+ 0.6)*  *(+ 0.7%)* | *328*  *(+ 2.3)*  *(+ 0.7%)* | *105*  *(- 0.4)*  *(- 0.4%)* | *70*  *(+ 2.1)*  *(+ 3.0%)* | *72*  *(+ 0.6)*  *(+ 0.8)* | *50*  *(+ 0.4)*  *(+ 0.7%)* | *32*  *(- 0.3)*  *(- 0.8)* |
| *Neoplasms lung cancer* | *83.3*  *(+ 0.7)*  *(+ 0.8%)* | *331*  *(+ 5.1)*  *(+ 1.6%)* | *106*  *(+ 0.5)*  *(+ 0.5%)* | *70*  *(+ 2.5)*  *(+ 3.7%)* | *72*  *(+ 1.0)*  *(+ 1.3%)* | *50*  *(+ 0.8)*  *(+ 1.6%)* | *32*  *(+ 0.4)*  *(+ 1.1%)* |
| *Neoplasms colorectal cancer* | *83.0*  *(+ 0.4)*  *(+ 0.4%)* | *328*  *(+ 1.9)*  *(+ 0.6%)* | *105*  *(- 0.7)*  *(- 0.7%)* | *69*  *(+ 1.7)*  *(+ 2.6%)* | *71*  *(+ 0.3)*  *(+ 0.5%)* | *50*  *(+ 0.4)*  *(+ 0.8%)* | *32*  *(+ 0.1)*  *(+ 0.4%)* |
| Mental and behavioral disorders | 83.2  (+ 0.5)  (+ 0.6%) | 268  (- 58.2)  (- 17.8%) | 89  (- 16.6)  (- 15.7%) | 32  (- 35.6)  (- 52.5%) | 69  (- 2.0)  (- 2.8%) | 48  (- 1.5)  (- 3.1%) | 30  (- 2.5)  (- 7.9%) |
| Infectious and parasitic disease | 82.8  (+ 0.1)  (+ 0.2%) | 319  (- 6.6)  (- 2.0%) | 105  (- 1.1)  (- 1.1%) | 68  (+ 0.5)  (+ 0.8%) | 69  (- 1.6)  (- 2.3%) | 47  (- 2.4)  (- 4.9%) | 30  (- 1.9)  (- 5.9%) |
| Endocrine, nutritional and metabolic | 83.0  (+ 0.4)  (+ 0.4%) | 321  (- 5.2)  (- 1.6%) | 104  (- 1.9)  (- 1.8%) | 68  (+ 0.2)  (+ 0.3%) | 70  (- 0.9)  (- 1.3%) | 47  (- 2.3)  (- 4.7%) | 32  (- 0.3)  (- 0.9%) |
| Diseases of the blood and the blood-forming organs | 82.7  (+ 0.0)  (+ 0.0%) | 325  (- 1.1)  (- 0.3%) | 105  (- 0.8)  (- 0.7%) | 68  (+ 0.1)  (+ 0.1%) | 71  (- 0.3)  (- 0.4%) | 49  (- 0.1)  (- 0.2%) | 32  (- 0.1)  (- 0.2%) |
| Diseases of the circulatory system | 85.6  (+ 2.9)  (+ 3.5%) | 324  (- 1.9)  (- 0.6%) | 98  (- 8.1)  (- 7.7%) | 76  (+ 8.5)  (+ 12.6%) | 71  (- 0.0)  (- 0.0%) | 47  (- 2.4)  (- 4.8%) | 32  (+ 0.1)  (+ 0.3%) |
| *Coronary Heart Disease* | *83.2*  *(+ 0.6)*  *(+ 0.7%)* | *326*  *(+ 0.6)*  *(+ 0.2%)* | *104*  *(- 2.0)*  *(- 1.9%)* | *71*  *(+ 3.1)*  *(+ 4.6%)* | *71*  *(+ 0.3)*  *(+ 0.4%)* | *49*  *(- 0.8)*  *(- 1.5%)* | *32*  *(- 0.0)*  *(- 0.0%)* |
| *Stroke* | *83.3*  *(+ 0.7)*  *(+ 0.8%* | *323*  *(- 3.3)*  *(- 1.0%)* | *101*  *(- 1.5)*  *(- 1.4%)* | *65*  *(- 3.1)*  *(- 4.5%)* | *71*  *(+ 0.4)*  *(+ 0.6%)* | *50*  *(+ 0.7)*  *(+ 1.5%)* | *32*  *(+ 0.1)*  *(+ 0.3%)* |
| Diseases of the nervous system | 83.0  (+ 0.3)  (+ 0.4%) | 307  (- 19.0)  (- 5.8%) | 99  (- 6.9)  (- 6.5%) | 67  (- 0.7)  (- 1.0%) | 68  (- 3.2)  (- 4.5%) | 42  (- 7.1)  (- 14.3%) | 31  (- 1.2)  (- 3.8%) |
| Diseases of the respiratory system | 83.5 (+ 0.9)  (+ 1.0%) | 323  (- 3.3)  (- 1.0%) | 102  (- 3.3)  (- 3.2%) | 71  (+ 3.7)  (+ 5.5%) | 70  (- 1.0)  (- 1.4%) | 47  (- 2.4)  (- 5.0%) | 32  (- 0.2)  (- 0.6%) |
| *COPD* | *83.0*  *(+ 0.4)*  *(+ 0.5%)* | *326*  *(- 0.4)*  *(- 0.1%)* | *105*  *(- 1.0)*  *(- 0.9%)* | *69*  *(+ 1.8)*  *(+ 2.7%)* | *71*  *(+ 0.0)*  *(+ 0.0%)* | *48*  *(- 1.2)*  *(- 2.5%)* | *32*  *(- 0.0)*  *(- 0.1%)* |
| Diseases of the digestive system | 83.0  (+ 0.4)  (+ 0.4%) | 305  (- 20.6)  (- 6.3%) | 100  (- 5.7)  (- 5.4%) | 69  (+ 1.5)  (+ 2.2%) | 59  (- 11.8)  (- 16.6%) | 46  (- 3.3)  (- 6.6%) | 31  (- 1.3)  (- 4.2%) |
| Diseases of the genitourinary system | 82.8  (+ 0.2)  (+ 0.2%) | 315  (- 10.4)  (- 3.2%) | 100  (- 5.3)  (- 5.0%) | 69  (+ 1.0)  (+ 1.4%) | 69  (- 1.7)  (- 2.4%) | 46  (- 3.5)  (- 7.0%) | 31  (- 0.9)  (- 2.8) |
| Pregnancy, childbirth and the puerperium | 82.7  (+ 0.0)  (+ 0.1%) | 314  (- 11.9)  (- 3.7%) | 101  (- 5.1)  (- 4.9%) | 68  (+ 0.0)  (+ 0.0%) | 66  (- 5.4)  (- 7.6%) | 49  (- 0.7)  (- 1.4%) | 31  (- 0.7)  (- 2.1%) |
| Diseases of the skin and subcutaneous tissue | 82.7  (+ 0.0)  (+ 0.0%) | 321  (- 4.6)  (- 1.4%) | 104  (- 2.0)  (- 1.9%) | 68  (- 0.1)  (- 0.1%) | 70  (- 1.4)  (- 2.0%) | 49  (- 0.9)  (- 1.8%) | 32  (- 0.3)  (- 0.9%) |
| Diseases of the musculoskeletal system and connective tissue | 82.7  (+ 0.1)  (+ 0.1%) | 300  (- 25.5)  (- 7.8%) | 96  (- 10.2)  (- 9.6%) | 66  (- 1.5)  (- 2.2%) | 62  (- 8.5)  (- 12.0) | 46  (- 3.7)  (- 7.5%) | 31  (- 1.6)  (- 5.0%) |
| Certain conditions in the perinatal period | 82.8  (+ 0.2)  (+ 0.2%) | 324  (- 2.0)  (- 0.6%) | 105  (- 1.3)  (- 1.3%) | 68  (+ 0.1)  (+ 0.2%) | 70  (- 0.5)  (- 0.7%) | 49  (+ 0.1)  (+ 0.2%) | 32  (- 0.3)  (- 1.0%) |
| Symptoms, signs and abnormalities | 83.0  (+ 0.4)  (+ 0.4%) | 300  (- 26.2)  (- 8.0%) | 96  (- 10.2)  (- 9.6%) | 69  (+ 1.1)  (+ 1.6%) | 61  (- 9.5)  (- 13.3%) | 43  (- 6.2)  (- 12.6%) | 31  (- 1.4)  (- 4.4%) |
| Injury, poison and others | 83.1  (+ 0.4)  (+ 0.5%) | 316  (- 9.8)  (- 3.0%) | 100  (- 5.9)  (- 5.6%) | 66  (- 1.4)  (- 2.1%) | 69  (- 2.2)  (- 3.2%) | 50  (+ 0.3)  (+ 0.7%) | 32  (- 0.5)  (- 1.6%) |
| *Traffic accident* | *82.7*  *(+ 0.1)*  *(+ 0.1%)* | *-* | *-* | *-* | *-* | *-* | *-* |

1. *Italics* and right text alignment indicate sub categories [↑](#footnote-ref-1)
